# To be or not to be [fertile], that is the question

**DOI:** 10.1186/s12610-016-0040-9

**Published:** 2016-10-12

**Authors:** N. Swierkowski-Blanchard, L. Alter, S. Salama, C. Muratorio, M. Bergere, M. Jaoul, F. Vialard, M. Bailly, J. Selva, F. Boitrelle

**Affiliations:** 1 Department of Reproductive Biology, Cytogenetics and Gynaecology, Poissy General Hospital, F-78303 Poissy, France; 2 EA7404-GIG, UFR des Sciences de la Santé Simone Veil, Université Paris-Saclay, Montigny le Bretonneux, France

**Keywords:** Spermatozoon, Male self-image, Male infertility, Azoospermia, Male feelings, Virility

## Abstract

**Background:**

According to our literature analysis, there are no data focused on spermatozoa emotional representations in childless men and data on the emotional repercussions of a diagnosis of infertility on men are still scarce. Thus, in this work, we investigated what the presence or absence of spermatozoa in the semen symbolize for men.

**Material and methods:**

To answer this question, 441 childless heterosexual men participated in an anonymous, prospective, Internet-based survey.

**Results:**

In response to the question “What would having a high or normal sperm count symbolize for you?” the most frequent answer was “ability to father a child”. Men living with a partner were significantly more likely than single men to answer “ability to father a child” (*p* < 0.05) and less likely to answer “virility” and/or “ability to have an erection/ejaculation” (*p* = 0.001). In response to the question “If you found out that you had a low sperm count or no spermatozoa at all, how would you feel?”, most of the men stated that they would be disappointed. Men living with a partner were more likely to state that they would feel ashamed (*p* < 0.05) or guilty with regard to their partner (*p* < 0.0001).

**Conclusions:**

These preliminary results should help us to improve (i) the way that male infertility is announced (it is easier to find the right words if one understands the possible importance of having a high sperm count) and (ii) the psychological, marital and sexual counselling provided to men with a diagnosis of infertility.

## Resume

**Contexte:**

Dans la littérature, peu d'articles traitent du ressenti des hommes vis à vis de leurs spermatozoïdes. Que signifie pour un homme “avoir ou non des spermatozoïdes”? Voilà la question que nous nous sommes posée.

**Materiel et methodes:**

Pour y répondre nous avons élaboré un questionnaire qui a été rempli en ligne par 441 hommes hétérosexuels âgés de 18 à 45 ans et sans enfants.

**Resultats:**

A la question, “que signifie pour vous avoir des spermatozoïdes?”, la majorité d’entre eux a répondu “être père”. Les hommes en couple ont statistiquement répondu plus fréquemment “être père” (*p* < 0.05) et significativement moins fréquemment “être un (vrai) homme”, “être viril”, “être capable d’avoir une érection/éjaculation” comparativement aux hommes célibataires (*p* = 0.001). A la question, “qu’ éprouveriez vous si on vous annonçait que vous n’aviez pas de spermatozoïdes ou moins que la normale?” la majorité d’entre eux à répondu “je serais déçu”. Les hommes en couples ont répondu significativement plus fréquemment qu’ils se sentiraient honteux (*p* < 0.05) ou coupables vis à vis de leur partenaire (*p* < 0.0001).

**Conclusions:**

Ces résultats préliminaires doivent nous aider à mieux comprendre le ressenti des hommes vis à vis de leurs spermatozoïdes et nous aider, nous spécialistes de l’infertilité, à mieux annoncer des infertilités par azoospermie ou oligospermie en adoptant une démarche de conseil psychologique, sexuel et conjugal dans l’annonce de cette infertilité masculine. En effet “ne pas avoir un nombre élevé de spermatozoïdes et a fortiori ne pas en avoir du tout” peut avoir un impact négatif sur l’homme en termes d’humeur, de culpabilité et d’estime de soi.

## Introduction

The spermatozoon was first visualized in 1687 by the Dutch scientist Antonie van Leuvenhoek, who initially considered it to be a parasite with no apparent function. The spermatozoon’s biological function has been since established but continues to be studied in greater detail. However, one can legitimately raise the following questions: what do spermatozoa mean to a man, and how do men feel about their spermatozoa? What does having a high or normal sperm count and conversely, what does having a low sperm count mean to men? To the best of our knowledge, there are no clear literature data on these questions in childless men who have never had a sperm analysis. Most literature studies have focused on the impact of infertility on women, and only a few have investigated the feelings of infertile men (for a review, see [1]). When we searched the PubMed database with the following search terms: “male infertility” and “psychological”: this research produced 1,064 journal articles published between September, 1954 and May, 2016. When we searched the PubMed database with the following search terms: “azoospermia” and “psychological”: this research produced “only” 49 journal articles published between February, 1965 and May, 2016. Among these studies, none answered to the questions we asked here.

Several studies have found that infertile couples are more likely to feel depressed, anxious and/or guilty than fertile couples. Accordingly, the European Society of Human Reproduction and Embryology (ESHRE) recently published guidelines on psychosocial care in infertility and medically assisted reproduction [2]. However, data on the emotional repercussions of a diagnosis of infertility on men are still scarce [3, 4]. It was shown that the announcement of male infertility can cause the same reactions as that of serious illness: shame [4–6], guilt, depression [1, 5–8], anxiety, stress [1, 7], decreased self-esteem and a desperate search for a cause. Furthermore, infertility may call a man’s sexual identity into question [9, 10]. A diagnosis of infertility may make a man feel unsatisfied with his life [11–13] and may even prompt suicidal feelings and strain in his marital, social and/or sexual relationships [7, 11, 12, 14–16] because of the close relationships between fertility and sexuality [15, 17–19].

Given that (i) there are no data focused on spermatozoa emotional representations in childless men and (ii) only a few authors have focused on the potential feelings experienced by childless men in whom infertility is diagnosed, we decided to survey men in the general population. Firstly, we explored the feelings that childless men have about a high or a low sperm count. Secondly, we asked them how they would feel if they were diagnosed with oligo- or azoospermia. We consider that better knowledge of childless men’s feelings about potential infertility may improve the provision of optimal psychological, sexual and medical care to infertile men.

## Material and methods

### The survey

The survey was open to heterosexual childless men aged between 18 and 45. The anonymous, online, French-language survey was performed via a survey website between September 2015 and March 2016. The study questionnaire had been designed by three andrologists and three specialists in sexual medicine. The survey was approved by the local independent ethics committee and men who filled questionnaires were informed and consent to publish have been obtained from the participants to report their data; there was a statement before beginning of the online questionnaire. The following six questions were asked:

1. How old are you? (answer in years).
2. Are you living with a partner? (answer: “yes” or “no”)
3. What would having a high or normal sperm count symbolize for you?

Multiple choice answers: (a) masculinity, virility (being a real man), (b) fatherhood (being able to father children), (c) well-being (being in good health), (d) ability to have an erection (if you have a high sperm count, you will be able to have an erection), (e) ability to ejaculate (if you have a high sperm count, you will be able to ejaculate), (f) ability to have an orgasm (if you have a high sperm count, you will be able to have an orgasm), (g) other feelings; please specify.

4) If you found out that you had a low sperm count or no spermatozoa at all, how would you feel?

Multiple choice answers: (a) nothing in particular, (b) satisfied, (c) ashamed, (d) disappointed, (e) angry, (f) guilty with regard to my partner, (g) other feelings; please specify.

5) How many times a week do you have sexual intercourse (answer: number of times)
6) Have you ever thought of having a sperm analysis (to find out your sperm count)? (answer: “yes” or “no”).

In case completion of the questionnaire prompted feelings of anxiety or stress, the e-mail address of one of the investigators (an andrologist and a specialist in sexual medicine) was given at the end of the questionnaire. Respondents could also contact an investigator if they had any queries regarding the questionnaire and its content. Because we hypothesized that answers to this survey could vary in function of religious or ethnical origins, we asked the survey website to include men, known as belonging to various religious or ethnical groups. Thus, the survey website ensured that the sample was representative of men in the French general population.

### Statistical analysis

Statistical analyses were carried out with SAS software (version 9.1, SAS Institute Inc., Cary, NC, USA). Data are expressed as the mean ± standard error of the mean (SEM) and [range]. For age and the frequency of sexual intercourse per week, two subgroups of participants (those living with a partner and those not) were compared using Student’s *t*-test. For all the other variables, the two groups were compared using a chi-squared test or (if the sample size was below five) Fisher’s test. The threshold for statistical significance was set to *p* <0.05 for all tests.

## Results

A total of 441 questionnaires were completed online. The participants’ mean age was 25.8 ± 0.3 [18-44], and the mean frequency of sexual intercourse per week was 2.5 ± 0.1 [0-10]. Numerous participants were living with a partner (*n* = 284, 64.4 %). The two subgroups (single men vs. those living with a partner) did not differ significantly in terms of age (26 ± 0.3 years vs. 25.5 ± 0.5 years old, respectively; *p* = 0.4). As expected, men living with a partner had sexual intercourse more frequently than single men did (3.0 ± 0.2 vs. 1.4 ± 0.1, respectively; *p* < 0.0001). Fifty-eight of the men (13.2 %) had already considered having a sperm analysis just to find out what his sperm count would be.

**Fig. 1:**
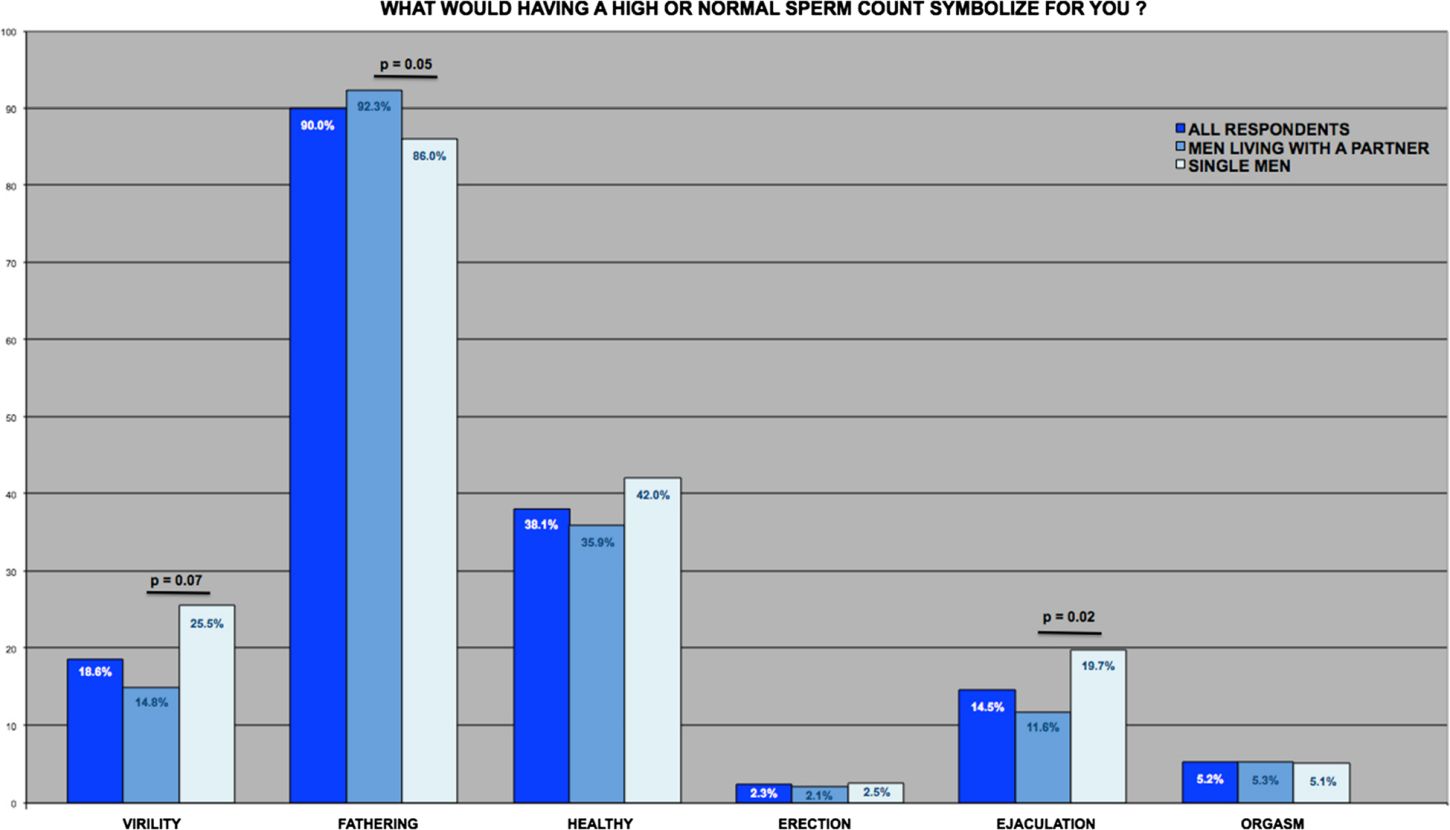
Proportion of the various answers to the question “what would having a high or normal sperm count symbolize for you?” for the 441 men included

**Fig. 2:**
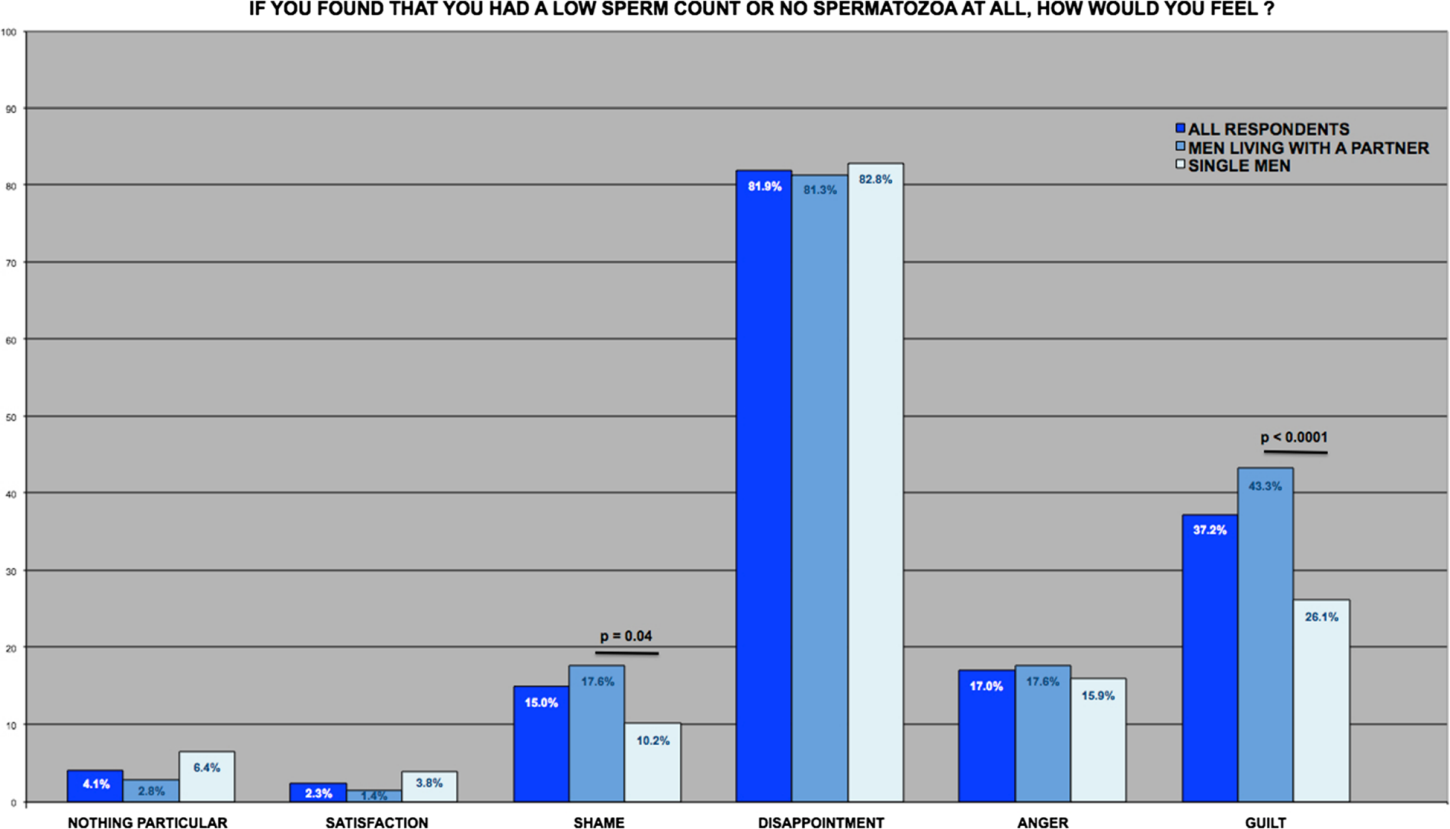
Proportion of the various answers to the question “If you found out that you had a low sperm count or no spermatozoa at all, how would you feel?” for the 441 men included

The data for questions 3 and 4 are shown in Figs. 1 and 2, respectively. In response to the question “what would having a high or normal sperm count symbolize for you?”, the most frequent answer (for 90 % of the respondents) was “being able to father a child”. A relatively high proportion (38.1 %) of the men also chose “being in good health”. Smaller proportions selected “virility” (18.6 %) and “ability to ejaculate” (14.5 %). Men living with a partner were significantly more likely than single men to answer “being able to father a child” (92.3 % vs. 86 %, respectively; *p* < 0.05) and significantly less likely to select “virility” (14.8 % vs. 25.5 %, respectively; *p* < 0.01) or “ability to ejaculate” (11.6 % vs. 19.7 %, respectively; *p* < 0.05). Overall, a relatively high proportion of the men (*n* = 124, 28.1 %) considered that having a high sperm count symbolized “virility” and/or “ability to have an erection” and/or “ability to ejaculate”. Men living with a partner were significantly less likely than single men to consider that having a high sperm count symbolized “virility” and/or “erection” and/or “ability to ejaculate” (*n* = 65 out of 284 (22.9 %) vs. *n* = 59 out of 157 (37.6 %), respectively; *p* = 0.001) and consider that having a normal or high sperm count symbolized good sexual health (*n* = 38 out of 284 (13.4 %) vs. *n* = 34 out of 157 (21.7 %), respectively; *p* = 0.02). None of the respondents chose the answer (g), i.e., “other feelings; please specify”.

In response to the question “If you found out that you had a low sperm count or no spermatozoa at all, how would you feel?”, most of the men (81.9 %) stated that they would be disappointed. A relatively high proportion of the men also stated also that they would feel guilty with regard to their partner (37.2 %). Lower proportions stated that they would feel angry (17.0 %) or ashamed (15.0 %). Men living with a partner were more likely than single men to state that they would feel ashamed (17.6 % vs. 10.2 %, respectively; *p* < 0.05) or guilty with regard to their partner (43.3 % vs. 26.1 %, respectively; *p* < 0.0001). None of the respondents chose the answer (g), i.e., “other feelings; please specify”.

Lastly, none of the respondents has emailed us for further information.

## Discussion

For the first time here, spermatozoa emotional representations in childless men and childless men’s feelings about potential infertility were described.

The survey’s first question was “What would having a high or normal sperm count symbolize for you?” As expected, 90 % of men considered that “having a normal or high sperm count” was associated with “being able to father a child”. We found that men responded “being a real man” and “ability to have an erection” and “ability to ejaculate”, more frequently than expected. The link between infertility and low self-esteem or between infertility and sexual disorders have already been described in infertile men. When infertility is diagnosed, the man may (i) feel that he is no longer a “real man” and (ii) develop sexual, erectile and/or ejaculatory dysfunctions [4, 7, 11, 15–21]. In contrast, the recent ESHRE guidelines concluded that patients in fertility work-up or initiating treatment with assisted reproductive technology (ART) do not have worse sexual relationships than men in the general population [2]. Our survey results suggest that men in the general population have a wide range of representations of their spermatozoa. For men in general (and particularly for single men), spermatozoa could symbolized “virility” and “good sexual health”. However, this does not necessarily mean that single men would develop sexual dysfunction if diagnosed as infertile. Likewise, we cannot affirm that men who considered spermatozoa to symbolize “virility” and “good sexual health” would be as sexually active if they were diagnosed as infertile. It is however important to note that links between virility or good sexual health and fertility exist. For the first time here, our survey results suggest that these links exist prior to a sperm analysis or diagnosis of infertility.

Secondly, we asked men how they would feel if diagnosed with oligo- or azoospermia. As expected (for a review, see [22]), the great majority of respondents (81.9 %) would be disappointed. Several men (more than we expected) also stated that they would feel guilty with regard to their partner (37.2 %). Compared with single men, men living with a partner were more likely to reply that they would feel ashamed or guilty with regard to their partner. These feelings were not mentioned in the ESHRE guidelines for patients diagnosed as infertile [2]. On the other hand, it was already shown that men may feel guilty about what their partner has to go through during ART (ovarian stimulation, oocyte retrieval, etc.) [9, 23]. Our results suggest that men diagnosed with infertility may feel disappointed, ashamed and guilty with regard to their partner - even before the initiation of an ART treatment programme. Our study suggested also that a small proportion of men would feel satisfied or would not feel anything in particular if they were told that they had few or no spermatozoa. This could be due to the fact that we did not ask the mean whether they wanted to have a child at some point in the future. Hence, we can simply hypothesize that men who stated that they would feel satisfied or would not feel anything in particular did not want to have a child at all.

Finally, even if the survey website ensured that the sample was representative of men in the French general population, it would be interesting in future research to assess social/religious/ethnic/cultural influences on these issues by adding a question (or more) about these social/religious/ethnic/cultural men’s characteristics. It would also be interesting to survey men undergoing a sperm analysis for the first time, in order to address these questions in men “really” diagnosed as infertile.

## Conclusions

Given that men often hide their feelings and suffering, it is important to know (i) the feelings that childless men have about a high or a low sperm count and (ii) what men might think and feel if diagnosed with oligo- or azoospermia. Knowing more about these issues should help us to (i) improve the way we announce male infertility (it is easier to find the right words when one knows what the presence of spermatozoa represents for a man) and (ii) improve the psychological, marital and sexual counselling of men diagnosed as infertile.

## Abbreviations

ART: Assisted reproductive technology
ESHRE: European Society of Human Reproduction and Embryology
SEM: Standard error of the mean
VS: Versus
